# Acetazolamide per os in Decompensated Chronic Heart Failure: Randomized Multicenter Trial ORION-A

**DOI:** 10.3390/jcm14186517

**Published:** 2025-09-16

**Authors:** Ibragim Sabirov, Olesya Rubanenko, Svetlana Villevalde, Anatoly Rubanenko, Nadezhda Veselovskaya, Vitaly Ivanenko, Natalia Kosheleva, Maksim Menzorov, Ilya Pochinka, Konstantin Protasov, Niyaz Khasanov, Sergey Yakushin, Elena Medvedeva, Dmitry Duplyakov

**Affiliations:** 1 Department of Therapy №2, Interstate Educational Organization of Higher Education Kyrgyz-Russian Slavic University Named After the First President of the Russian Federation B.N. Yeltsin, Bishkek 720000, Kyrgyzstan; 2 Department of Hospital Therapy with Courses in Hematology and Transfusiology, Samara State Medical University, Samara 443099, Russia; 3 Almazov National Medical Research Centre, Saint Petersburg 197341, Russia; 4 Department of Propaedeutic Therapy with the Course of Cardiology, Samara State Medical University, Samara 443099, Russia; 5 Department of Cardiology and Cardiovascular Surgery, Altai State Medical University, Barnaul 656038, Russia; 6 Volgograd Regional Clinical Cardiology Center, Volgograd 400008, Russia; 7 Department of Hospital Therapy, Saratov State Medical University Named After V.I. Razumovsky, Saratov 410053, Russia; 8 Department of Therapy and Occupational Diseases, Ulyanovsk State University, Ulyanovsk 432017, Russia; 9 Department of Endocrinology and Internal Diseases, Privolzhsky Research Medical University, Nizhny Novgorod 603006, Russia; 10 Russian Medical Academy of Continuing Professional Education, Irkutsk 664049, Russia; 11 Department of Propaedeutics of Internal Diseases Named After Professor S.S. Zimnitsky, Kazan State Medical University, Kazan 420012, Russia; 12 Department of Hospital Therapy with a Course of Medical and Social Expertise, Ryazan State Medical University, Ryazan 390026, Russia

**Keywords:** chronic heart failure, congestion, decompensation, acetazolamide, diuretic therapy

## Abstract

**Background/Objectives**: Overcoming resistance to diuretics is extremely important in decompensated chronic heart failure (HF). The objective of this study was to assess the efficacy and safety of oral acetazolamide, in addition to standard therapy, in HF patients admitted to the hospital with decompensation requiring intravenous loop diuretic therapy. **Methods**: In this open-label, prospective, multicenter, randomized trial, we included 416 patients hospitalized with decompensated HF. The patients were randomized into two groups: (1) standard therapy, and (2) standard therapy + acetazolamide orally 250 mg 3 times a day in the first 3 days of hospitalization. At randomization, oral thiazide/thiazide-like and loop diuretics were stopped, and intravenous furosemide was initiated. **Results**: Successful decongestion within 72 h of randomization was observed in 82 patients (39.6%) in the acetazolamide group and in 83 patients (39.7%) in the standard therapy group (*p* = 0.983). There was a significant difference in the increase in diuresis in the first 72 h (*p* = 0.028) and in natriuresis on the 2nd day (*p* = 0.04). There were no differences between the groups in duration of stay in the intensive care unit, duration of index hospitalization, 6 min walk test distance, and clinical assessment scale scores. Death from any cause occurred in three (1.4%) patients in the acetazolamide group, and in the same number of patients in the standard therapy group (*p* = 0.996). Death from cardiovascular cause and due to decompensated HF also did not differ between the groups during follow-up. **Conclusions**: The addition of acetazolamide to standard therapy in decompensated chronic HF resulted in a higher cumulative urine output during the first 72 h and natriuresis on the 2nd day after randomization.

## 1. Introduction

Decompensation of heart failure (HF) is a serious burden for healthcare worldwide. It is associated with hospitalizations and changes in the prognostic trajectory for most patients, and requires significant economic costs not only at the hospital stage but also for outpatient monitoring after discharge [1].

Evaluation of congestion in patients with decompensated HF is possible due to tools such as physical examination, clinical scales, biomarkers, imaging, and hemodynamic and impedance techniques [2]. Pathophysiological mechanisms of congestion in HF are multifaceted: systolic and/or diastolic dysfunction, sodium and water imbalance, sympathetic nervous system activation, endothelial damage, changes in venous capacity, and interstitial properties [3]. Diuretic therapy is the cornerstone of effective decongestion; its use in combination with optimal guideline-directed medical therapy (GDMT) promotes a complex effect on different pathogenic links of decompensation and congestion. However, the use of loop diuretic therapy is often not associated with complete elimination of congestion symptoms during hospitalization, which is explained by the limited effectiveness of loop diuretics and the development of resistance to them.

The addition of acetazolamide, an inhibitor of renal tubular carbonic anhydrase, may improve the efficacy of loop diuretics and potentially enhance decongestion. In prospective randomized trials (ADVOR [4], DIURESIS-HF [5]), the addition of parenteral acetazolamide to a loop diuretic was associated with higher diuresis and natriuresis in acute decompensated HF. However, in several countries, only the tablet form of acetazolamide is registered, and the results of these RCTs cannot be extended to its use.

The problem of resistance to diuretic therapy is particularly discussed in patients with acute HF; European guidelines [6] recommend the diuretic therapy algorithm with the option of using acetazolamide in combination with loop diuretics. For patients with decompensated chronic HF, the problem of overcoming resistance to diuretics is also extremely relevant but has not been sufficiently studied.

The open-label, prospective, multicenter, randomized trial ORION-A (ClinicalTrials.gov, NCT05802849) assessed the efficacy and safety of acetazolamide in tablet form in addition to standard therapy in patients with decompensated chronic HF.

## 2. Materials and Methods

**Figure 1:**
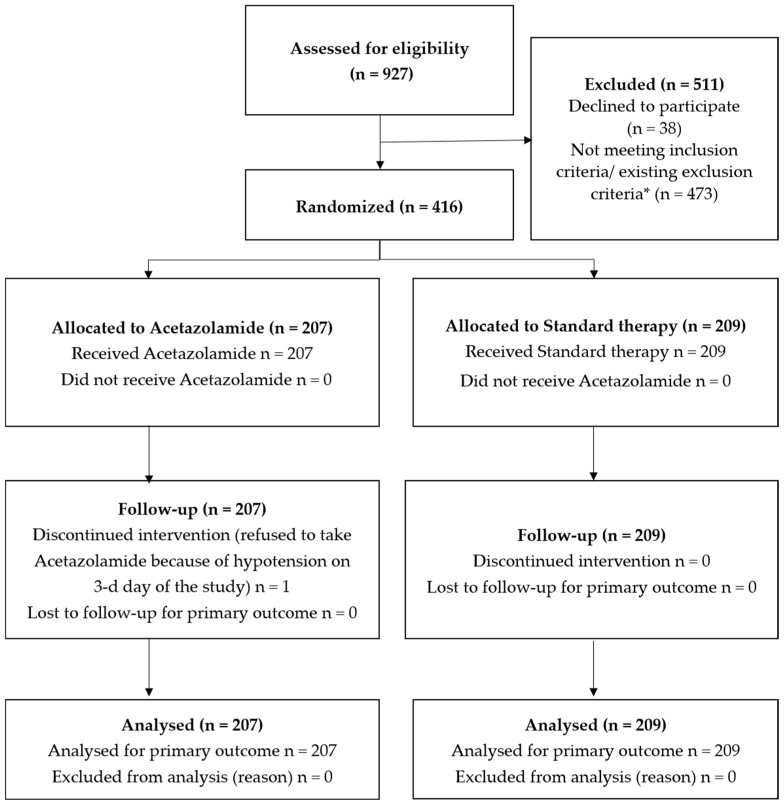
ORION-A trial flow diagram. * Not meeting inclusion criteria (n = 266): insufficient signs of volume overload, which are not required intravenous diuretics—166 patients, natriuretic peptides below thresholds—100 patients; exclusion criteria exist (n = 207): acetazolamide therapy within one month prior to admission—17 patients, hypokalemia—18 patients, hyponatremia—2 patients, systolic blood pressure < 90 mmHg—45 patients, emergency conditions—42 patients, history of dialysis or creatinine clearance < 10 mL/min—23 patients, decompensated diabetes mellitus—35 patients, malignant neoplasm in the phase of active treatment or terminal form of cancer—8 patients, severe anemia (hemoglobin < 7.0 g/dL)—17 patients.

ORION-A is an open, multicenter, block-randomized investigator-initiated study; the protocol has been published previously [7]. This research was carried out according to the CONSORT 2025 guidelines [8]. It included 416 patients from 12 centers hospitalized with decompensated chronic HF between April 2023 and October 2024 (Figure 1). Each patient was assigned a personal code in accordance with the randomization list. The random allocation sequence was generated by O.R. Patients were randomized into 2 groups: (1) standard therapy, and (2) standard therapy + acetazolamide orally 250 mg 3 times a day in the first 3 days of hospitalization. This study was conducted in accordance with the standards of good clinical practice and the ethical requirements of the Declaration of Helsinki of the World Medical Association. All patients signed an informed consent form before inclusion in this study.

### 2.1. Study Population

Patients admitted to the cardiology department with decompensation of chronic HF, were screened in this study. Diagnosis of HF was confirmed according to ESC guidelines [6]. According to the trial protocol, adult patients with chronic HF with any ejection fraction admitted to the hospital with decompensation requiring intravenous loop diuretic therapy were included in this study. All inclusion and exclusion criteria are presented in Table 1.

### 2.2. Study Design

**Figure 2:**
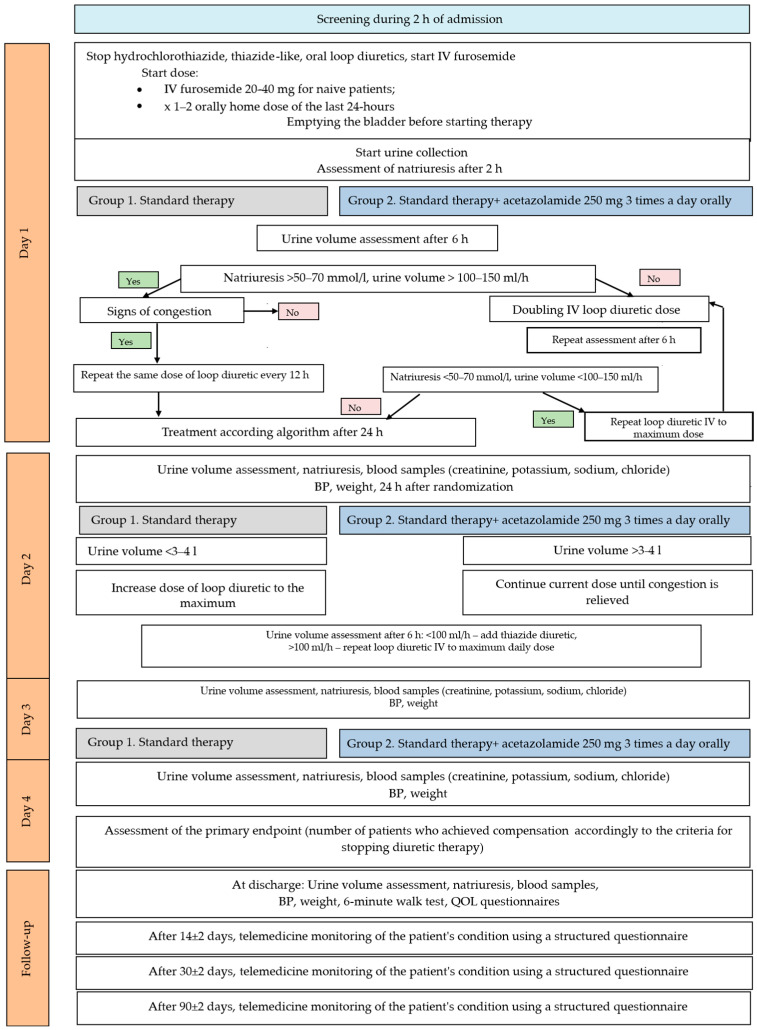
Study design. BP—blood pressure, IV—intravenous, QOL—quality of life.

Patients were screened within 24 h of admission. All patients underwent transthoracic echocardiography, chest radiography, and standard laboratory testing at the screening stage. Eligible patients were enrolled in this study. The study design is presented in Figure 2. Patients were randomized into 2 groups by generating random numbers: (1) standard therapy, and (2) standard therapy with the addition of acetazolamide orally 250 mg 3 times a day during 72 h. The follow-up period was 90 days.

### 2.3. Study Intervention

At randomization, oral thiazide/thiazide-like and loop diuretics were stopped, and intravenous furosemide was initiated. The initial dose of intravenous furosemide was determined based on the daily dose before hospitalization. If the patient was not taking oral loop diuretics, the furosemide dose was 20–40 mg intravenously; if the patient was taking them, the patient received intravenous furosemide at the same or double the oral maintenance dose for the last 24 h. Further tactics of intravenous diuretic therapy were determined depending on natriuresis and/or urine output, as well as the presence of symptoms of congestion (Figure 1). After 2 h, natriuresis was assessed; after 6 h, the volume of excreted urine was assessed. If natriuresis > 50–70 mmol/L and/or urine output > 100–150 mL/h, congestion symptoms were assessed. In the absence of symptoms, other causes of dyspnea were considered taking into account the rapid relief of congestion. If symptoms of congestion persisted, the intravenous dose of diuretic was repeated every 12 h. If natriuresis < 50–70 mmol/L, and urine volume < 100–150 mL/h, the dose of intravenous diuretic was doubled, and urine volume and/or natriuresis were re-evaluated after 6 h. If after 6 h natriuresis < 50–70 mmol/L, and urine output < 100 mL/h, intravenous diuretic was repeated with an increase in the dose to the maximum. If, after 6 h, natriuresis > 50–70 mmol/L, and urine volume > 100–150 mL/h, treatment was carried out according to the algorithm after 24 h. On the 2nd day, if the urine volume was <3–4 L, the furosemide dose was increased to the maximum and, after 6 h, diuresis was assessed; if it was lower than 100 mL/h, thiazide diuretics were added (Figure 2).

### 2.4. Urinary Collections and Blood Samples

Before starting intravenous administration of loop diuretics, the patient was advised to empty the bladder (if this was not possible, bladder catheterization was performed). Urine collection began immediately after administration of intravenous furosemide or furosemide in combination with oral acetazolamide. Special care was taken to ensure that all urine is collected.

Venous blood was collected every morning at 8:00 a.m. for the 3 days following randomization and before discharge. Creatinine, potassium, sodium, chloride, urea, and acid-base balance were determined. NT-proBNP and BNP were determined in plasma by methods used in routine practice at the study centers.

A venous blood sample was collected fasting before the first intravenous administration of furosemide and was repeated every morning at 8:00 a.m. for the next 3 days and before discharge. Creatinine, potassium, sodium, chloride and urea were assessed. The plasma levels of NT-proBNP and BNP were measured using methods of routine practice at the study centers.

### 2.5. Study Endpoints

The primary endpoint included the number of patients who achieved compensation according to the criteria for stopping diuretic therapy (the number of patients who scored 0–1 points on the congestion scale) within 72 h after randomization.

Secondary endpoints were as follows: (1) increase in urine output during the first 72 h of hospitalization (from the time of randomization), (2) weight loss, (3) natriuresis (estimated in 24 h urine), (4) duration of hospital stay, (5) duration of stay in the intensive care unit, (6) death from any cause within 90 days, (7) death from cardiovascular cause within 90 days, (8) death from HF decompensation within 90 days, (9) the number of pleural and pericardial punctures performed during hospitalization, and (10) 6 min walk test distance at discharge.

### 2.6. Safety Monitoring

During this study, the following adverse events were systematically monitored: (1) doubling of serum creatinine level from baseline, (2) renal replacement therapy, (3) hypokalemia (serum potassium < 3.5 mmol/L), (4) hypotension (systolic blood pressure < 90 mm Hg), (5) metabolic acidosis, and (6) syncope.

### 2.7. Statistical Analysis

The sample size calculation to test superiority was based on the expected difference in effects (42.2% of patients in the acetazolamide group were compensated with diuretic therapy, while in the group without acetazolamide, it was 30.5%). The power of this study was 80% with a *p*-value of <0.05. Superiority analyses were conducted on an intention-to-treat basis (participants who fulfilled eligibility were randomly assigned to either of the study drugs and had data on the outcome of interest as pre-defined in our protocol).

A statistical analysis was performed with the use of IBM SPSS (version 26). Medians and interquartile ranges were used for data presentation. Missing data were not replaced. Comparison of quantitative variables between the two groups was performed using the Mann–Whitney U-test. Categorical data are presented as percentages and compared with the Chi-squared test. Statistical significance was set at a two-tailed probability level of <0.05.

## 3. Results

### 3.1. Study Population

A total of 416 patients were randomized to receive either standard therapy (209 participants) or standard therapy + acetazolamide (207 participants). The baseline characteristics of patients are presented in Table 2. The groups were comparable in terms of the main clinical and demographic parameters. The patients of the standard therapy group had a higher incidence of ischemic heart disease (161 (77%) vs. 140 (67.6%), *p* = 0.032) and lower baseline estimated glomerular filtration rate (eGFR) (*p* = 0.021) compared with the acetazolamide group; they were comparable in other comorbidities and laboratory parameters. The groups did not differ in the representation of congestion scale components (edema, pleural effusion, ascites), HF phenotypes according to ejection fraction, or NT-proBNP and BNP levels (Table 2).

Despite the absence of requirements for standard therapy (prescribed in accordance with real clinical practice), a large proportion of patients in both groups received renin–angiotensin system (RAS) inhibitors (85.2% vs. 85%), beta-blockers (93.8% vs. 89.9%), mineralocorticoid receptor antagonist (MRA) (89.5% vs. 88.4%) and sodium–glucose cotransporter 2 (SGLT2) inhibitors (73.2% vs. 76.3%). The dose of intravenous loop diuretic (furosemide) was similar in the two trial groups. Thus, the groups were comparable in terms of HF therapy, including quadruple therapy and diuretic therapy (Table 2).

### 3.2. Primary Endpoints

Successful decongestion within 72 h from randomization was observed in 82 patients (39.6%) in the acetazolamide group and in 83 patients (39.7%) in the standard therapy group (*p* = 0.983).

### 3.3. Secondary Endpoints

There was a significant difference in the increase in diuresis in the first 72 h (*p* = 0.028) and in natriuresis on the 2nd day (*p* = 0.04) (Table 3).

There were no differences between the groups in duration of stay in the intensive care unit, duration of index hospitalization, or the number of pleural and pericardial punctures performed during hospitalization (Table 3).

Secondary endpoints assessed at hospital discharge (6 min walk test distance and clinical assessment scale scores) were also comparable in both groups (Table 3).

Death from any cause occurred in three (1.4%) patients in the acetazolamide group, and in the same number of patients in the standard group (*p* = 0.996). Death from cardiovascular cause and due to decompensated HF also did not differ between the groups during follow-up (Table 3).

### 3.4. Safety and Adverse Events

The incidence of adverse events was similar in the two trial groups (Table 4). Severe metabolic acidosis was not observed in either group during the treatment phase. None of the patients required renal replacement therapy.

## 4. Discussion

In this multicenter randomized trial involving 416 patients with decompensated chronic HF, the addition of acetazolamide to standard therapy was not associated with a higher incidence of successful decongestion in the 3 days after randomization. However, acetazolamide therapy resulted in a significant increase in urine volume during the first 72 h and increase in natriuresis on the 2nd day compared with standard therapy. Acetazolamide therapy was safe and was not associated with severe adverse events. The incidence of adverse events registered in the acetazolamide group was comparable to that in the standard treatment group. The acetazolamide group did not differ from the standard therapy group in terms of duration of hospital stay and duration of treatment in the intensive care unit. At discharge, the groups were also comparable in clinical parameters, and the follow-up period revealed no differences in mortality rates.

Acetazolamide is a well-known drug, used not only in cardiology but also in neurological and ophthalmological practice, and included in World Health Organization model list of essential medicines [9]. A renaissance of interest in acetazolamide for the correction of congestion in HF has occurred in the last decade. The efficacy of acetazolamide in addition to loop diuretic therapy in patients with decompensated HF was assessed in a meta-analysis that included three randomized controlled trials [10]. Only one of the studies, involving twenty patients, assessed the effects during the period of decompensated chronic HF [11], while the others were devoted to acute HF [4,5]. Thus, ORION-A is the first relatively large study to assess the efficacy of acetazolamide in patients with decompensated chronic HF. The results of this study, on the one hand, confirm the hypothesis of increased diuretic effect during acetazolamide therapy, and on the other hand, highlight a number of questions for further study and evaluation.

ORION-A did not demonstrate superiority in decongestion in the acetazolamide group compared with standard care, as found in the ADVOR trial but not in the DIURESIS-HF trial, which used intravenous acetazolamide in acute decompensated HF. Two pilot studies using oral acetazolamide in acute HF [12] and decompensated chronic HF [11] did not assess decongestion.

The lack of difference in decongestion between groups may be partly due to the use of oral acetazolamide, which has potentially lower bioavailability than the intravenous form because of the intestinal wall edema during decompensation. A multicenter study comparing intravenous and oral acetazolamide in patients with chronic HF and diuretic-induced metabolic alkalosis found that the intravenous form had advantages in the primary endpoint (reduction in serum bicarbonate after 24 h) [13]. The oral form of acetazolamide is characterized by imperfect physicochemical properties, including low solubility, and modern research is being conducted to improve the bioavailability of the tablet using various technologies, including co-crystals [14].

In our trial, a significant effect on cumulative diuresis after 72 h and natriuresis on day 2 was obtained. At the same time, in the study by T. Imiela et al. with oral acetazolamide, there was not significant effect on natriuresis, but a difference in fluid balance and severity of dyspnea was observed [11]. Thus, in patients with a long duration of chronic HF and different experiences with diuretic therapy, longer treatment with acetazolamide may be required for effective decongestion and to overcome resistance to loop diuretics.

The guideline-recommended medical therapy can significantly improve the prognosis of HF patients. SGLT2 inhibitors are an important component of such therapy, effective in patients of all ejection fraction categories [15]. The previously mentioned pilot study with acetazolamide in decompensated chronic HF [11] was conducted before recommendations for the use of SGLT2 inhibitors, and the ORION-A trial demonstrates effects with the use of this class of drugs in 76.3% of patients in the acetazolamide group and 73.2% in the standard therapy group (*p* = 0.46). Moreover, the results of our study indicate the safety of using acetazolamide and SGLT2 inhibitors simultaneously, which is important for practical use.

In the “Great Debate”, the lack of an effect on hard endpoints was identified as one of the barriers to the use of acetazolamide [16]. Indeed, acetazolamide had no effect on death during the first 3 months in both acute HF trials and in the present study of decompensated HF. However, the aim of its use is to overcome resistance to diuretic therapy and reduce congestion, while another tool, GDMT, improves prognosis and prevents decompensation.

Despite the available data, there are still many questions about diuretic therapy in patients with decompensated HF. Various candidate markers are currently being studied to evaluate the efficacy and safety of diuretic therapy. The PUSH-AHF trial demonstrated the superiority of natriuresis-guided diuretic therapy over standard care in patients with acute HF [17]. Evaluation of natriuresis in the ORION-A study was not a mandatory condition for determining the tactics of diuretic therapy; at the same time, this method is an accessible and promising tool in real clinical practice. In a small pilot study by A. Kosiorek et al. [12], in addition to standard laboratory tests, such urinary biomarkers as neutrophil gelatinase lipocalin, cystatin-C, and kidney injury molecule-1 were assessed to monitor the safety of acetazolamide and loop diuretic therapy, but the usefulness of such monitoring requires confirmation. The further development of guided personalized diuretic therapy using an available arsenal of diuretics, including acetazolamide, seems relevant.

## 5. Study Limitations

This trial has several limitations: (1) the majority of patients who participated in the trial were White, and this study also included patients of the Kyrgyz ethnic group, which may limit the generalizability of our results to other racial or ethnic groups; (2) unblinded study design; (3) assessment of natriuresis was optional according to the protocol and was performed in 50% of patients; (4) the congestion scale used to assess the primary endpoint reflects mainly extracellular volume overload; and (5) outpatient therapy was performed in a real-life clinical setting.

## 6. Conclusions

In this randomized multicenter trial, we found that the addition of acetazolamide to standard therapy, including intravenous loop-diuretic in patients with decompensated chronic HF, led to a significant increase in urine volume during the first 72 h and an increase in natriuresis on the 2nd day after randomization.

## Figures and Tables

**Table 1:** Eligibility criteria for ORION-A trial.

**Inclusion Criteria**
(1) emergency hospital admission with decompensated chronic HF II–IV NYHA, requiring intravenous diuretics; (2) adults > 18 years; (3) any left ventricular ejection fraction (LVEF), in patients with LVEF ≥ 50%: presence of cardiac structural abnormalities and/or diastolic dysfunction and/or increased plasma levels of BNP > 400 pg/mL or NT-proBNP > 450 pg/mL for patients under 50 years, >900 pg/mL and >1800 pg/mL for age groups of 50–75 years and >75 years, respectively.
**Exclusion Criteria**
(1) acetazolamide therapy within one month prior to admission; (2) expected use of intravenous inotropes, vasopressors, or sodium nitroprusside at any time during the study; (3) exposure to nephrotoxic agents (i.e., contrast dye) anticipated within the next 3 days; (4) hypersensitivity to acetazolamide, other sulfonamides, and/or drug ingredients; (5) systolic blood pressure < 90 mmHg; (6) pregnancy and breastfeeding; (7) hypokalemia (potassium < 3.5 mmol/L); (8) hyponatremia (sodium < 135 mmol/L), (9) severe chronic renal failure (creatinine clearance < 10 mL/min) or renal replacement therapy/ultrafiltration at any time before this study; (10) emergency conditions (myocardial infarction, stroke, pulmonary embolism, acute myocarditis, pericarditis); (11) liver cirrhosis with encephalopathy and liver failure, (12) metabolic acidosis; (13) severe anemia (hemoglobin < 7.0 g/dL); (14) acute renal failure; (15) Addison’s disease; (16) decompensated diabetes mellitus; (17) congenital heart disease; (18) malignant neoplasm in the phase of active treatment or terminal form of cancer; (19) hypocortisolism.

**Table 2:** Baseline characteristics of the groups.

Characteristic	Standard Therapy Acetazolamide (−) n = 209	Standard Therapy Acetazolamide (+) n = 207	*p*
**Clinical and demographic parameters**			
Age, years	68 (61–74)	67 (61–73)	0.732
Male sex, n (%)	128 (61.2%)	133 (64.3%)	0.526
Race/ethnicity			0.865
White, n (%)	160 (76.6%)	157 (75.8%)	
Asian, n (%)	50 (24.2%)	49 (23.4%)	
Smoking, n (%)			0.788
Current	39 (18.7%)	42 (20.3%)	
No	136 (65.1%)	134 (64.7%)	
Ex-smokers	31 (14.8%)	26 (12.6%)	
Unknown	3 (1.4%)	5 (2.4%)	
Weight, kg	88 (77.0–98.2)	90 (78.0–103.0)	0.214
Body mass index, kg/m^2^	30.1(27.6–34.6)	31.1 (27.2–35.7)	0.372
Functional class NYHA, n (%)			0.732
II	37 (17.7%)	29 (14.0%)	
III	108 (51.7%)	108 (52.2%)	
IV	64 (30.6%)	70 (33.8%)	
LVEF, %	40.0 (30.0–51.0)	40.0 (30.0–53.5)	0.753
HFpEF, n (%)	58 (27.8%)	64 (30.9%)	0.674
HFmrEF, n (%)	40 (19.1%)	34 (16.4%)	
HFrEF, n (%)	111 (53.1%)	109 (52.7%)	
Components of congestion score, n (%)			
Edema	170 (81.3%)	175 (84.5%)	0.386
Pleural effusion	65 (31.1%)	73 (35.2%)	0.426
Ascites	52 (24.9%)	55 (26.6%)	0.736
Systolic blood pressure, mm Hg	121 (111–144)	122 (113–132)	0.374
Diastolic blood pressure, mm Hg	82 (73–94)	84 (73.0–82.5)	0.229
**Laboratory parameters**			
NT-proBNP, pg/mL	1534.0 (612.8–3592.5)	1445.5 (713.0–3139.5)	0.702
BNP, pg/mL	580 (186–1557)	692 (195–1777)	0.385
eGFR, mL/min/1.73 m^2^	54 (43–68)	60 (47–71)	0.021
Hemoglobin, g/dL	13.4 (12.0–14.6)	13.0 (11.8–14.4)	0.345
Sodium, mmol/L	140 (138–142)	141 (138–143)	0.102
Potassium, mmol/L	4.39 (3.90–4.80)	4.40 (4.00–4.75)	0.812
Natriuresis, mmol/L	110.00 (76.28–128.50)	115.65 (91.23–144.86)	0.098
Diuresis, mL	2100 (1800–2500)	2100 (1600–2500)	0.867
**Comorbidities, n (%)**			
Hypertension	196 (93.8%)	186 (89.9%)	0.144
Stroke history	28 (13.4%)	17 (8.2%)	0.089
Ischemic heart disease	161 (77.0%)	140 (67.6%)	0.032
Atrial fibrillation/flutter	126 (60.3%)	116 (56%)	0.380
Diabetes mellitus	32 (15.3%)	27 (13%)	0.507
Chronic kidney disease			
C3	116 (55.5%)	95 (45.9%)	0.050
C4	12 (5.7%)	9 (4.3%)	0.671
COPD	44 (21.1%)	38 (18.4%)	0.490
Treatment *, n (%)			
ACE inhibitor	101 (48.3%)	104 (50.2%)	0.696
ARB	30 (14.4%)	21 (10.1%)	0.191
ARNI	47 (22.5%)	51 (25.6%)	0.439
Beta-blockers	196 (93.8%)	186 (89.9%)	0.372
Mineralocorticoid receptor antagonist	187 (89.5%)	183 (88.4%)	0.728
SGLT2 inhibitors	153 (73.2%)	158 (76.3%)	0.463
Quadruple therapy			
Overall	121 (57.9%)	115 (55.6%)	0.630
HFpEF	20 (34.5%)	22 (34.4%)	0.990
HFmrEF	20 (50.0%)	15 (44.1%)	0.613
HFrEF	81 (73.0%)	78 (71.6%)	0.815
Dose of furosemide *, mg	40 (40–60)	40 (40–60)	0.143
Dose of furosemide, mg (day 2)	60 (40–80)	80 (40–100)	0.403
Dose of furosemide, mg (day 3)	80 (40–80)	80 (40–100)	0.450
CRT **, n (%)	1 (0.5%)	3 (1.4%)	0.371
ICD **, n (%)	3 (1.4%)	5 (2.4%)	0.501

*—after randomization (day 1), **—before inclusion in this study, Missing data: NT-proBNP—85 (20.4%), BNP—321 (77.2%), natrium—2 (0.5%), potassium—1 (0.2%). ACE—angiotensin-converting enzyme, ARB—angiotensin receptor blocker, ARNI angiotensin receptor–neprilysin inhibitor, BNP—brain natriuretic peptide, COPD—chronic obstructive pulmonary disease, CRT—cardiac resynchronization therapy, GFR glomerular filtration rate, HFpEF—heart failure with preserved ejection fraction, HFrEF—heart failure with reduced ejection fraction, HFmrEF—heart failure with mildly reduced ejection fraction, ICD—implantable cardioverter-defibrillator, LVEF—left ventricular ejection fraction, NT-proBNP—N-terminal pro–B-type natriuretic peptide, SGLT-2—sodium–glucose cotransporter 2.

**Table 3:** Secondary endpoints.

Parameter	Standard Therapy Acetazolamide (−) n = 209	Standard Therapy Acetazolamide (+) n = 207	*p*
Increase in the volume of urine excreted in the first 72 h of hospitalization (from the time of randomization), mL	5825 (4826–6926)	6060 (5225–7335)	0.028
Weight loss within 72 h, kg (delta)	3.00 (2.00–5.00)	3.00 (2.00–5.10)	0.897
Weight loss during hospitalization, kg (delta)	4.00 (3.00–7.25)	4.00 (2.45–8.05)	0.816
Natriuresis (mmol/L):			
Day 2	101.25 (73.96–119.80)	113.40 (90.40–140.80)	0.004
Day 3	95.41 (68.69–120.00)	105.00 (63.83–123.94)	0.640
Day 4	95.00 (63.74–111.25)	100.00 (65.36–113.06)	0.403
At the time of discharge	90.52 (72.11–105.00)	70.66 (58.85–104.02)	0.806
Duration of hospital stay, days	4.00 (4.00–9.00)	4.00 (4.00–8.00)	0.820
Duration of stay in intensive care unit, days	3.00 (3.00–4.00)	4.00 (3.00–4.00)	0.156
Number of pleural and pericardial punctures performed during hospitalization, n (%)	2 (1.0%)	0 (0%)	0.999
Assessment of clinical statement score, points	4.00 (2.5–7.00)	4.00 (2.00–7.00)	0.632
6 min walk test at discharge, m	309.50 (282.50–404.50)	300.00 (214.50–347.00)	0.250
Death from any cause within 90 days, n (%)	3 (1.4%)	3 (1.4%)	0.996
Death from cardiovascular cause within 90 days	1 (0.5%)	1 (0.5%)	
Death from heart failure decompensation within 90 days	0 (0%)	2 (1%)	

Missing data: natriuresis day 2—208 (50%), natriuresis day 3—217 (52.2%), natriuresis day 4—217 (52.2%), natriuresis at the time of discharge—218 (52.4%), weight loss during hospitalization—4 (1%), duration of stay in intensive care unit—294 (70.7%), duration of hospital stay—1 (0.5%), death—82 (19.7%).

**Table 4:** Incidence of adverse events during treatment phase and index hospitalization.

Adverse Events	Standard Therapy Acetazolamide (−) n = 209	Standard Therapy Acetazolamide (+) n = 207	*p*
**During treatment phase—n (%)**			
Doubling of serum creatinine level from baseline	0 (0%)	1 (0.5%)	0.999
Renal replacement therapy	0 (0%)	0 (0%)	-
Hypotension (SBP < 90 mm Hg)	2 (1.0%)	3 (1.4%)	0.644
Hypokalemia	28 (13.4%)	29 (14.0%)	0.918
Severe metabolic acidosis	0 (0%)	0 (0%)	-
Syncope	0 (0%)	0 (0%)	-
**During hospitalization phase—** **n (%)**			
Doubling of serum creatinine level from baseline	0 (0%)	1 (0.5%)	0.999
Renal replacement therapy	0 (0%)	0 (0%)	-
Hypotension (SBP < 90 mm Hg)	2 (1.0%)	4 (1.9%)	0.400
Hypokalemia	36 (17.2%)	37 (17.9%)	0.862
Severe metabolic acidosis	0 (0%)	0 (0%)	-
Syncope	0 (0%)	0 (0%)	-

eGFR—estimated glomerular filtration rate, SBP—systolic blood pressure.

## Data Availability

The original materials presented in this trial are included in this article/Supplementary Material; further inquiries can be directed to the corresponding authors.

## Supplementary Materials

The following supporting information can be downloaded at: https://www.mdpi.com/article/10.3390/jcm14186517/s1, Supplementary Materials: The questionnaire for telemedicine visits.

## Abbreviations

The following abbreviations are used in this manuscript:

ACE	Angiotensin-converting enzyme
ARB	Angiotensin receptor blocker
ARNI	Angiotensin receptor–neprilysin inhibitor
BNP	Brain natriuretic peptide
COPD	Chronic obstructive pulmonary disease
CRT	Cardiac resynchronization therapy
eGFR	Estimated glomerular filtration rate
GDMT	Guideline-directed medical therapy
HF	Heart failure
ICD	Implantable cardioverter-defibrillator
HFmrEF	Heart failure with mildly reduced ejection fraction
HFpEF	Heart failure with preserved ejection fraction
HFrEF	Heart failure with reduced ejection fraction
LVEF	Left ventricular ejection fraction
MRA	Mineralocorticoid receptor antagonist
NT-proBNP	N-terminal pro–B-type natriuretic peptide
SBP	Systolic blood pressure
SGLT2	Sodium–glucose cotransporter 2

## Appendix A

**Table A1:** The investigators of ORION-A trial.

Investigators’ Names	Affiliations
Abdilazizova E.A.	Interstate Educational Organization of Higher Education Kyrgyz-Russian Slavic University named after the first President of the Russian Federation B.N. Yeltsin, Bishkek, Kyrgyz Republic
Amirova Yu.I., Vdovina S.V., Gabidullova D.A., Emelyanova N.G., Kornyakova N.I., Ponomar I.A., Sinkevich O.V., Sheshunova E.M.	Samara Regional Clinical Cardiology Dispensary named after V.P. Polyakov, Samara, Russia
Budkina M.L.	Privolzhsky Research Medical University, Nizhny Novgorod, Russia
Demeshchenko E.A.	Volgograd Regional Clinical Cardiology Center, Volgograd, Russia
Ivantsov E.N.	Kazan State Medical University, Kazan, Russia
Lazareva D.V.	Ryazan regional clinical cardiology dispensary, Ryazan, Russia
Nurgaziewa D.S.	Regional Clinical Hospital, Saratov, Russia
Pereverzeva K.G., Pravkina E.A.	Ryazan State Medical University, Ryazan, Russia
Podusov A.S., Troshina N.V., Shafigulin A.R.	Central Medical and Sanitary Unit named after Honored Doctor of Russia V.A. Egorov, Ulyanovsk, Russia
Fedorishina O.V.	Russian Medical Academy of Continuing Professional Education, Irkutsk, Russia
Frolova E.S.	Altai regional cardiological dispensary, Barnaul, Russia
Shlyakova A.A.	City Clinical Hospital No. 13 of the Avtozavodsky District of Nizhny Novgorod, Nizhny Novgorod, Russia
Yagudina R.N.	Irkutsk City Clinical Hospital No. 3, Irkutsk, Russia
