# Supplementary material for: Acetazolamide per os in Decompensated Chronic Heart Failure: Randomized Multicenter Trial ORION-A
Source: J Clin Med. 2025 Sep 16;14(18):6517. doi: 10.3390/jcm14186517 (PMC12470467; doi:10.3390/jcm14186517)
Supplement: Supplementary file 1 [file jcm-14-06517-s001.zip › jcm-3869330-supplementary.pdf]

Supplementary Materials

Questionnaire

Telemedicine visit (30+/-2 days after discharge from hospital)

Contact with the patient or his legal representative _____yes_____no

During the observation period: Death ______yes (date)_________no__________unknown

Cause: Cardiovascular_________ Other reason_____________

Ambulance Calls ______yes_________no Number of calls___________

Hospitalization for cardiovascular disease____yes______no

Hospitalization for other diseases (except CVD)____yes_________no

Cause ____

Increasing shortness of breath______yes_________no

Increasing edema ______yes_________no

Bleeding episode ______yes_________no

Body weight ___________kg

Heart rate __________ beats/min SBP ______/ DBP ______ mmHg (lying down, sitting)

Treatment

Statins ____specify drug + dose

SGLT2i ____specify drug + dose

ACE inhibitors ____specify drug + dose

ARBs____specify drug + dose

ARNI (Valsartan + Sacubitril) ___________________________specify drug + dose

Beta-blockers ____specify drug + dose

Mineralocorticoid receptor antagonists____________________ specify drug + dose

Ivabradine ____ dose

Digoxin____ dose

Diuretics____ specify drug + dose

Acetazolamide/Indapamide/ Chlorthalidone/Hydrochlorothiazide/ Furosemide/ Torasemide/others

Antiplatelet therapy____________________________________specify drug + dose

Anticoagulants____specify drug + dose

Antidiabetic drugs______________________________________specify drug+ dose

Iron supplements_______________________________________specify drug+ dose

Telemedicine visit (90+/-2 days after hospital discharge)

Contact with the patient or his legal representative _____yes_____no

During the observation period: Death ______yes (date)_________no__________unknown

Cause: Cardiovascular_________ Other ______________

Ambulance Calls ______yes_________no   Number of calls___________

Hospitalization for cardiovascular disease____yes______no

Hospitalization for other diseases (except CVD)___yes_________no

Cause ____

Increasing shortness of breath______yes_________no

Increasing edema ______yes_________no

Bleeding episode ______yes_________no

Body weight ___________kg

Heart rate __________ beats/min   SBP ______/ DBP ______ mmHg (lying down, sitting)

Treatment

Statins ____specify drug + dose

SGLT2i ____specify drug + dose

ACE inhibitors _______________________________________specify drug + dose

ARBs____specify drug + dose

ARNI (Valsartan + Sacubitril) ___________________________specify drug + dose

Beta-blockers ____specify drug + dose

Mineralocorticoid receptor antagonists____________________ specify drug + dose

Ivabradine ____ dose

Digoxin____ dose

Diuretics____ specify drug + dose

Acetazolamide/Indapamide/ Chlorthalidone/Hydrochlorothiazide/ Furosemide/ Torasemide/others

Antiplatelet therapy___________________________________specify drug + dose

Anticoagulants_______________________________________specify drug + dose

Antidiabetic drugs____________________________________specify drug+ dose

Iron supplements_____________________________________specify drug+ dose
